# Renal cell carcinoma in autosomal dominant polycystic kidney disease: A case report

**DOI:** 10.1016/j.radcr.2023.09.011

**Published:** 2023-10-04

**Authors:** Gullyawan Rooseno, Ida Bagus Gde Tirta Yoga Yatindra, Wahjoe Djatisoesanto, Tarmono Djojodimedjo

**Affiliations:** a Department of Urology, Faculty of Medicine, Airlangga University, Surabaya, East Java, Indonesia; b Dr. Soetomo General-Academic Hospital, Surabaya, East Java, Indonesia

**Keywords:** Renal cell carcinoma, Multi cystic kidney, Case report, Incidental findings

## Abstract

Autosomal dominant polycystic kidney disease (ADPKD) is one of the congenital cystic renal diseases with the highest incidence. ADPKD was suspected of being a risk factor for the emergence of RCC. A 65-year-old male complained of numbness in both knees for a week. The patient came to a neurosurgeon and was advised to perform a lumbosacral MRI. The patient had no complaints. The patient had a history of hypertension but was never treated. Computed tomography intravenous pyelogram (CT-IVP) revealed a heterogeneous lobulated mass in the upper to the middle of the right kidney to the right renal hilus. It also revealed multiple cysts, in both kidneys. The patient underwent an open radical nephrectomy in the right kidney with minimal bleeding. Three years revealed no pain at the surgery site or hematuria. Abdominal MRI revealed no recidive mass. This case report comprehensively described an autosomal dominant polycystic kidney disease that coexists with RCC. malignant lesions were found in ADPKD cases without any clinical symptoms of malignancy. M malignant lesions could be discovered by chance in nephrectomy specimens. Autosomal dominant polycystic kidney disease with renal cell carcinoma is a unique presentation. Despite the rarity of the situation, the patient was successfully treated.

## Introduction

Autosomal dominant polycystic kidney disease (ADPKD) is one of the congenital cystic renal diseases with the highest incidence (1:500-1:1000). Autosomal dominant polycystic kidney disease is associated with changes in ciliary function caused by PKD 1 and PKD 2 gene mutations. Walters and Braasch, in 1934, suggested a suspicion of ADPKD as a predisposing factor to the emergence of RCC [[1], [2], [3], [4]]. However, until now, this theory has been debated due to the limited number of studies.

Renal cell carcinoma (RCC) is a kidney malignancy that accounts for 5% of male and 3% of female cancer cases globally. Recently, the number of RCCs has increased due to incidental findings during the radiologic gastrointestinal examination or musculoskeletal problems. There were 140,000 deaths worldwide related to RCC each year. RCC ranks 13th for malignancy-related mortality [5]. This case report aims to describe an incidental finding of RCC in ADPKD patients.

## Case presentation

A 65-year-old male complained of numbness in both knees for a week. The patient came to a neurosurgeon and was advised to perform a lumbosacral MRI. The patient had no complaints regarding low back pain, hematuria, or masses in the abdomen. There was no family history of malignancy or bilateral kidney cysts. The patient had a history of hypertension but was never treated. Lumbosacral MRI results incidentally revealed a solid mass with a necrotic component in the right kidney with a size of 6.4 × 5.5 cm.

**Fig. 1:**
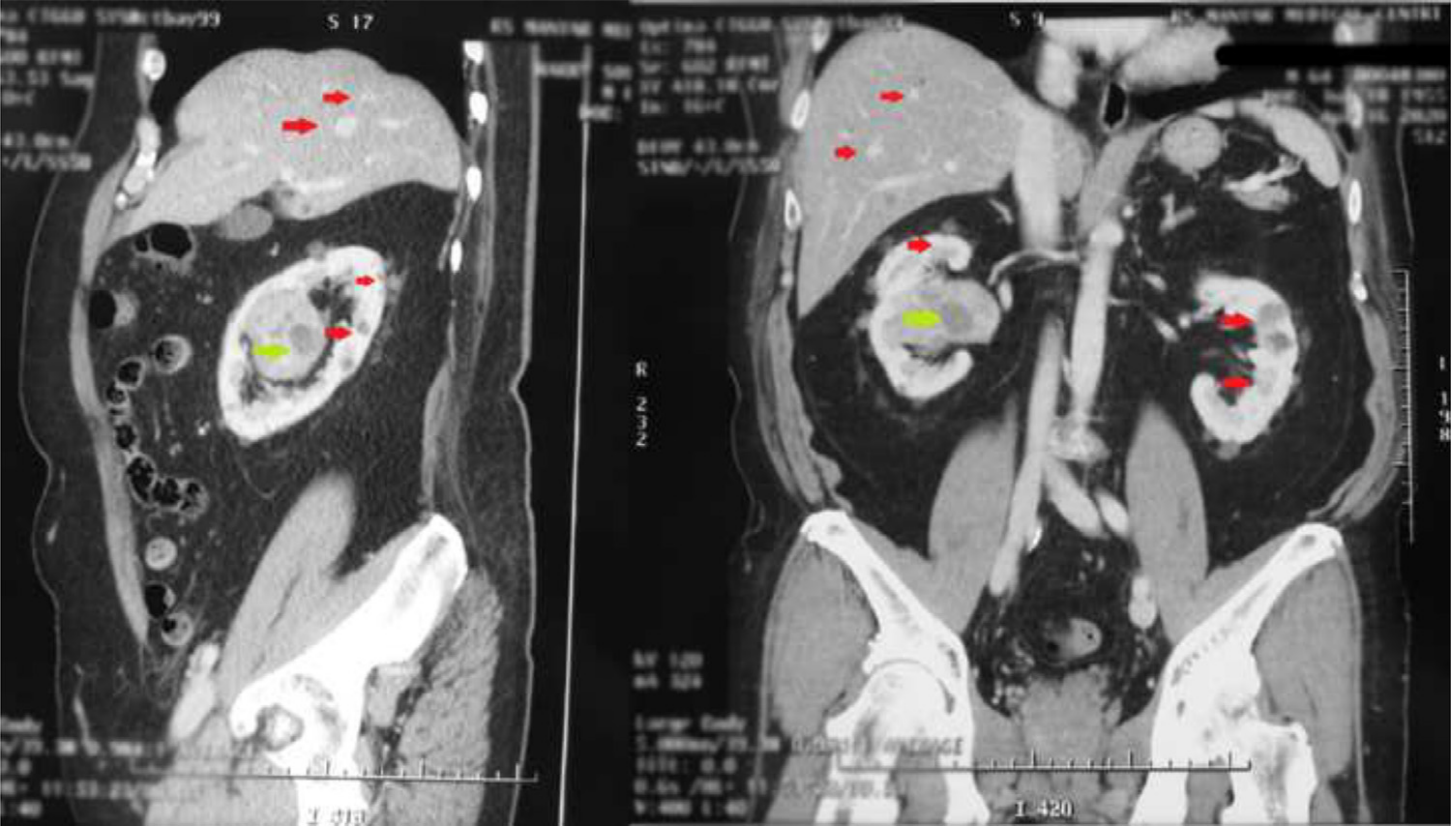
The results of the CT-IVP examination revealed a lobulated heterogeneous mass in the upper to middle pole of the right kidney, accompanied by multiple cysts on the right and left kidneys (green arrow). Multiple cysts were found in the right and left lobes of the liver (red arrow).

A Physical examination revealed a blood pressure of 150/90 mm Hg. There was no mass palpated on the patient's flank. Computed tomography intravenous pyelogram (CT-IVP) revealed a heterogeneous lobulated mass in the upper to middle of the right kidney to the right renal hilus with a size of 6.4 × 5.2 × 5.5 cm compressing the upper and middle calyx and renal pelvis of the right kidney. There was contrast enhancement on the mass. Multiple cysts of sizes 0.5-2.5 cm were revealed in both kidneys. In addition, numerous nonenhancing cysts were found in the right and left lobes of the liver, with a size of 0.5-2 cm (Fig. 1).

**Fig. 2:**
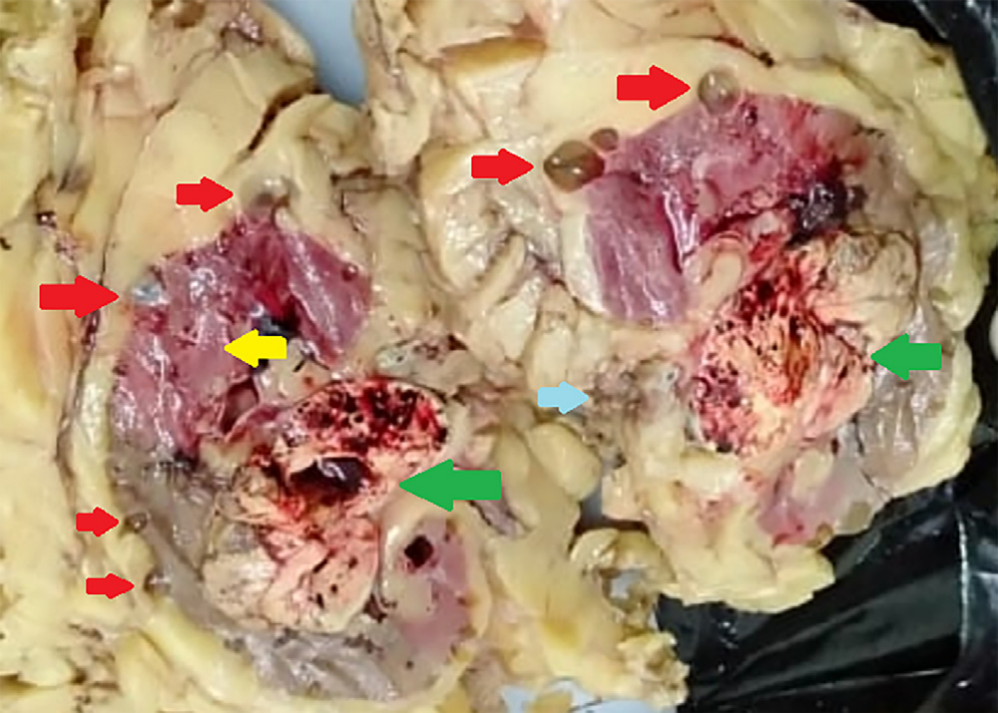
Macroscopic appearance of renal cell carcinoma. Yellowish fragile tumors are indicated by the green arrow; multiple cystic cavities adhering to perirenal fat are indicated by the red arrow; the yellow arrow indicates normal renal parenchyma remnants; and the blue arrow indicates renal vessels with no infiltration.

**Fig. 3:**
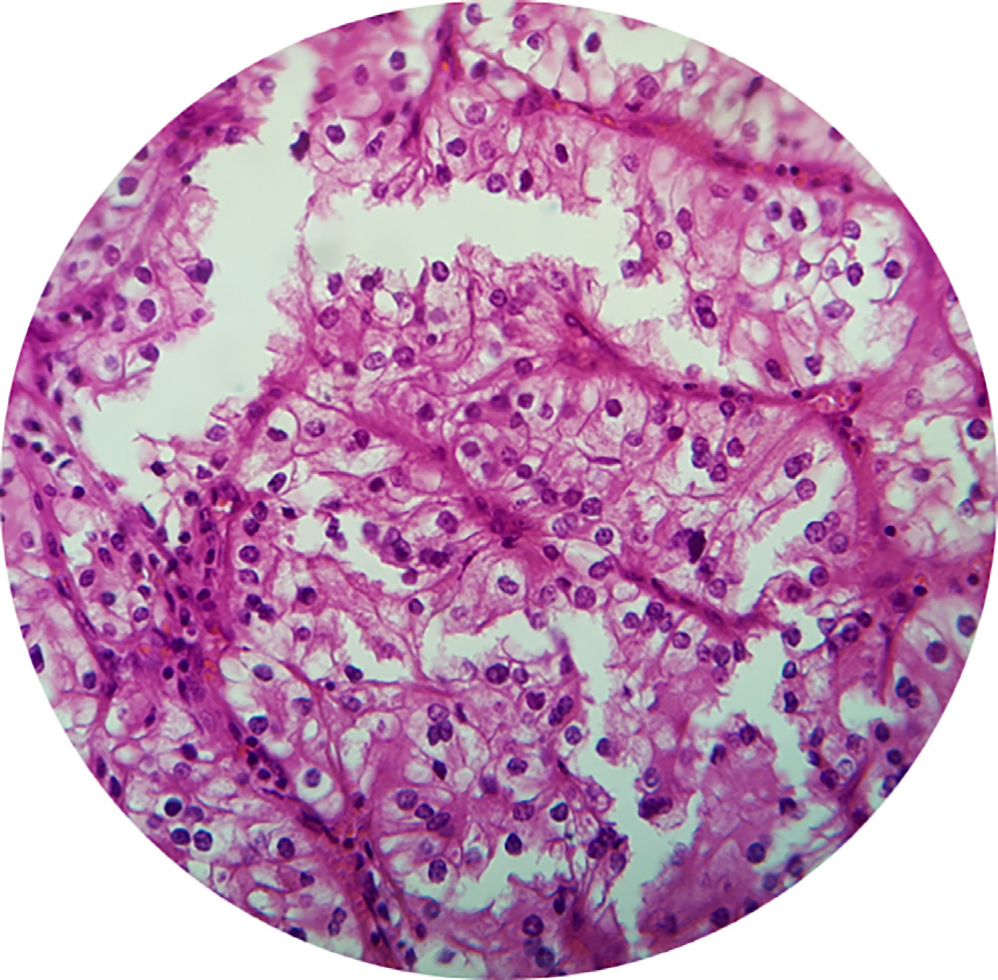
Microscopic appearance of renal cell carcinoma. The tumor shows an alveolar structure of clear anaplastic cells and many thin-walled blood vessels.

The patient was suspected of having a malignant right kidney tumor accompanied by ADPKD. The patient underwent an open radical nephrectomy in the right kidney. It was found that a kidney with a size of 10 × 5 × 4 cm was attached to the perirenal fat by cystic formations on the surface of the kidney capsule. A tumor was found with slightly cystic characteristics, nodular, soft, and loose with bleeding in a smaller cyst with a size of 6 × 5 × 4 cm. No thrombus or tumor material was found in the renal vessels (Fig. 2). Histopathology examination of open granular chromatin with 40 x magnification found the alveolar structure of a clear anaplastic cell and many thin-walled blood vessels. The cytoplasmic was eosinophilic or clear, nuclear grade II, with conspicuous (prominent) red nucleoli. It was concluded that the tumor type was RCC clear cell type: grade II, and the pathological stage of the tumor was pT3 (Fig. 3).

**Fig. 4:**
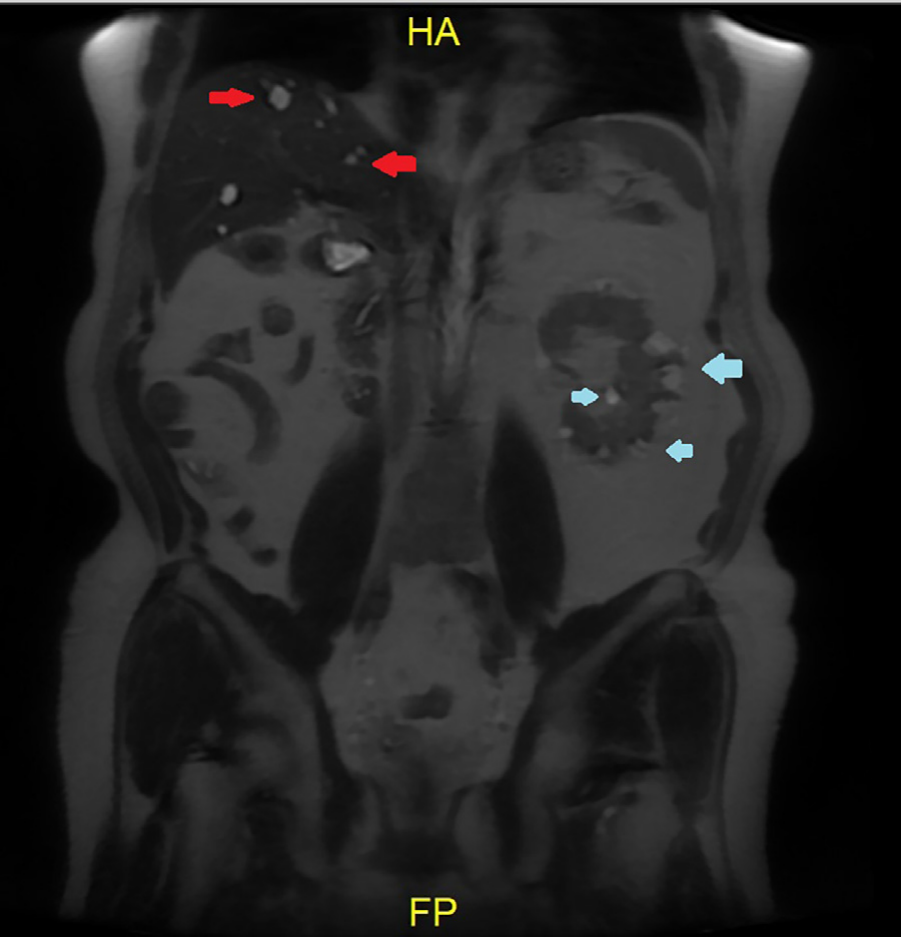
Follow-up MRI revealed multiple cysts on the liver (red arrow) and kidney (blue arrow) with the largest diameter of 2 cm and no sign of a residive mass.

Follow-up 3 years after surgery revealed no pain at the surgery site or hematuria and the numbness on both knees has disappeared. The patient was generally in good condition and could return to normal activities. The abdominal MRI results revealed no recidive mass at the surgical bed, there are multiple cysts in the liver and left kidney, and there are no signs of vertebral metastasis. The creatinine serum level was 1.31 mg/dL (Fig. 4).

## Discussion

Autosomal dominant polycystic kidney disease is a congenital disorder of the kidneys characterized by 2 unilateral or bilateral cysts before the age of 30 years, at least 2 cysts on each kidney between the ages of 30 and 59 years, or 4 cysts on each kidney at age 60 years or older. Apart from the number of cysts obtained, patients with ADPKD may also have associated cysts in the liver or pancreas. In a study conducted by Hajj et al. [2], the incidence of ADPKD was twice as high in men as in women.

The diagnosis of ADPKD in this patient was based on the diagnostic criteria described by Grantham et al. [4], which are more than 4 kidney cysts in each kidney in patients over 60 years old and accompanied by liver or pancreatic cysts. In several studies, malignant lesions were found in ADPKD cases without any clinical symptoms of malignancy. Malignant lesions were usually discovered incidentally in nephrectomy specimens [5,6]. The prevalence of RCC in ADPKD patients was 8.73% (7/84 kidneys) [2]. A retrospective study conducted by Jilg et al. [3] reported a higher incidence of ADPKD accompanied by malignant lesions in male patients (11 patients out of a total of 173 patients with ADPKD who were operated on) (6.35%).

Surgery would be the mainstay of treatment for localized RCC in ADPKD, either partial nephrectomy for those with normal renal function or radical nephrectomy, frequently bilateral, for those with end-stage renal disease on dialysis. Chemotherapy or immunotherapy can be given to patients with metastatic disease. mTOR inhibitors have also been found to be effective in metastatic RCC, either alone or in combination with VEGF-targeted medications [7]. The patient in this case was managed with radical nephrectomy.

The prognosis of ADPKD with RCC remains unclear, and the high incidence of RCC found in ADPKD patients in earlier research may simply be the result of end-stage renal disease (ESRD) brought on by ADPKD [6]. Compared to clear cell RCC, papillary RCC is considered to have a better prognosis [7]. Sarcomatoid RCC has the worst prognosis [6].

ADPKD is suspected to be associated with impaired ciliary function (ciliopathy). This condition is caused by a mutation of polycystin-1, which is a protein produced by the PKD 1 gene, and polycystin-2, which is a protein produced by the PKD 2 gene. The mutation in PKD 1 and PKD 2 was found in 15% of cases [1]. Along with kidney cysts, ADPKD patients frequently also develop cysts in other organs. The liver is the most commonly affected organ. Although liver cysts often do not interfere with normal liver function, they can occasionally hemorrhage, rupture, become infected, or result in symptoms like abdominal discomfort [8]. However, there is no abdominal pain in this patient.

In this case, the patient had a history of uncontrolled hypertension. A meta-analysis of 18 prospective studies and 14 case-control studies showed a relationship between hypertension and an increased risk of RCC [9]. Every 10 mm Hg increase in blood pressure increases the risk of RCC by 10%-22% [10]. The biological mechanism for this relationship is still unclear. Some researchers hypothesized that chronic renal hypoxia and lipid peroxidation were responsible for reactive oxygen species (ROS) formation, which contributes to RCC development [5]. However, Hidayat et al. reported that hypertension and RCC share a number of similar risk factors, which emphasizes the need for adequate correction for these confounding variables during the evaluation of the causal relationship between hypertension and RCC. It might be challenging to discern the order in which RCC and hypertension arise if RCC is identified in individuals with hypertension [10]. It might be possible that the cause of this patient's hypertension was not related to the RCC but to the degenerative disease, as the patient was 60 years old.

## Conclusion

Autosomal-dominant polycystic kidney disease is a rare disease, especially when it occurs concurrently with RCC. In this case, multiple RCCs in ADPKD were found incidentally during radiology and histopathology examination of nephrectomy specimens, even though there was no clinical sign of malignancy. The suggested treatment for RCC with ADPKD is radical nephrectomy. Routine follow-up every 6 months for 2 years, then yearly with an abdominal CT scan, chest X-ray, and blood chemistry analysis, is recommended by the European Society of Medical Oncology.

## Patient consent

Informed consent for patient information to be published in this article was obtained.
